# Arginylation Regulates G-protein Signaling in the Retina

**DOI:** 10.3389/fcell.2021.807345

**Published:** 2022-01-21

**Authors:** Marie E. Fina, Junling Wang, Pavan Vedula, Hsin-Yao Tang, Anna Kashina, Dawei W. Dong

**Affiliations:** ^1^ Department of Biomedical Sciences, School of Veterinary Medicines, University of Pennsylvania, Philadelphia, PA, United States; ^2^ Proteomics and Metabolomics Facility, The Wistar Institute, Philadelphia, PA, United States; ^3^ Institute for Biomedical Informatics, Perelman School of Medicine, University of Pennsylvania, Philadelphia, PA, United States

**Keywords:** arginylation, G-protein signaling, RGS, retina, mass spectrometry

## Abstract

Arginylation is a post-translational modification mediated by the arginyltransferase (Ate1). We recently showed that conditional deletion of Ate1 in the nervous system leads to increased light-evoked response sensitivities of ON-bipolar cells in the retina, indicating that arginylation regulates the G-protein signaling complexes of those neurons and/or photoreceptors. However, none of the key players in the signaling pathway were previously shown to be arginylated. Here we show that G*α*t1, G*β*1, RGS6, and RGS7 are arginylated in the retina and RGS6 and RGS7 protein levels are elevated in Ate1 knockout, suggesting that arginylation plays a direct role in regulating their protein level and the G-protein-mediated responses in the retina.

## 1 Introduction

Arginylation is a post-translational modification that has been implicated in a large number of key physiological processes (see, e.g., Zanakis et al., 1984; Bongiovanni et al., 1999; Kwon et al., 2002; Hu et al., 2005; Karakozova et al., 2006; Decca et al., 2007; Rai et al., 2008; Saha and Kashina, 2011; Lee et al., 2012; Jiang et al., 2016; Wang et al., 2017a; Wang et al., 2017b). It was initially characterized as a post-translational modification of the protein N-termini for ubiquitin-proteasomal degradation (Ciechanover et al., 1988; Balzi et al., 1990). N-terminal arginylation can target proteins for degradation both *in vitro* (Davydov and Varshavsky, 2000) and *in vivo* (Lee et al., 2005) via ubiquitin-proteasome dependent N-end rule pathway (Varshavsky, 2011). While initially arginylation was believed to be exclusively targeting the N-terminal Asp, Glu, or Cys, as well as Asn and Gln upon deamidation, it has been later shown that arginylation can also target internal Asp and Glu residues by conjugating Arg to their acidic side chains (Wang et al., 2014). This discovery expands the scope of potentially arginylated proteins.

G-protein signaling is involved in virtually every known physiological process and plays a major role in neural signal transduction (Stewart and Fisher, 2015; Squires et al., 2018). It involves three crucial components: G-protein coupled receptors (GPCRs), G-protein heterotrimers, and Regulator of G-protein signaling (RGS) proteins. Upon stimulation, GPCRs facilitate the formation of G*α*-GTP and promote the dissociation of G*α* and G*βγ* from each other and from GPCRs. Opposite to GPCRs, RGS proteins accelerate the termination of G-protein signaling by facilitating hydrolysis of the GTP bound to the G*α* subunit of the G-protein, and thus promoting its re-association with G*βγ* subunits and GPCRs (De Vries et al., 1995; Dohlman et al., 1995; Druey et al., 1996; Hunt et al., 1996; Koelle and Horvitz, 1996; Watson et al., 1996).

We recently discovered that conditional deletion of arginyltransferase (Ate1) in the nervous system affects G-protein signaling in the retina. In particular, lack of Ate1 leads to increased light-evoked response sensitivities of ON-bipolar cells, as well as their downstream neurons (Fina et al., 2021), indicating that arginylation might be involved in the three G-protein signaling complexes of the first two stages of visual signal transduction. Among the GPCRs, G-proteins, and RGSs in the mouse retina (see Table 1), RHO, G*β*5L, G*γ*13, and RGS11 have the N-end rule residues, while others have the potential to be arginylated on the side chains. However, none of them are known to be arginylated.

**TABLE 1:** Components of the G-protein signaling complexes in the retina screened for arginylation. The coverage is calculated with the MS/MS peptides identified during tryptic/P search against the whole mouse proteome without including arginylation into the search. The depth is the average number of MS/MS scans of the identified peptides over each covered amino acid (AA) base. Rgs9 (short) isoform and Gnb5 (long) isoform are listed. The latter encodes two protein isoforms: G*β*5 and G*β*5L. The three well-known G-protein signaling complexes in the retina are: RHO-G*α*t1-G*β*1-G*γ*t1-RGS9-G*β*5L, OPN1MW/OPN1SW-G*α*t2-G*β*3-G*γ*2-RGS9-G*β*5L, mGluR6-G*α*o-G*β*3-G*γ*13-RGS7/RGS11-G*β*5.

Gene name	AA length	Coverage(%)	depth	protein symbol	protein name
					G-proteins
Gnao1	354	63	131	G*α*o	G-protein Go subunit alpha
Gnat1	350	75	373	G*α*t1	G-protein Gt subunit alpha-1
Gnat2	354	64	132	G*α*t2	G-protein Gt subunit alpha-2
Gnb1	340	74	285	G*β*1	G-protein Gi,s,t subunit beta-1
Gnb3	340	67	53	G*β*3	G-protein Gi,s,t subunit beta-3
Gng2	71	72	11	G*γ*2	G-protein Gi,s,o subunit gamma-2
Gng13	67	84	7	G*γ*13	G-protein Gi,s,o subunit gamma-13
Gngt1	74	84	225	G*γ*t1	G-protein Gt subunit gamma-T1
					G-protein coupled receptors
Grm6	871	19	2	mGluR6	Metabotropic glutamate receptor 6
Opn1mw	359	19	6	OPN1MW	Medium-wave-sensitive opsin 1
Opn1sw	346	29	16	OPN1SW	Short-wave-sensitive opsin 1
Rho	348	32	202	RHO	Rhodopsin
					GTPase accelerating proteins
Gnb5	395	56	31	G*β*5(L)	G-protein subunit beta-5
Rgs6	472	47	11	RGS6	Regulator of G-protein signaling 6
Rgs7	469	28	13	RGS7	Regulator of G-protein signaling 7
Rgs9	484	75	23	RGS9	Regulator of G-protein signaling 9
Rgs11	443	2.9	2	RGS11	Regulator of G-protein signaling 11

Here we performed mass spectrometry analysis of mouse retina, using both our own data and data from the public domain, to investigate if any of the proteins involved in G-protein signaling in the retina are arginylated. We found that G*α*t1, G*β*1, RGS6 and RGS7 were arginylated. Immunostaining revealed a prominent increase in the levels of RGS6 and its obligatory binding partner G*β*5 in synaptic processes of starburst amacrine cells of Ate1 knockout retina, corroborating our previous finding that RGS7 and G*β*5 increase in synaptic processes of ON-bipolar cells. We propose that arginylation regulates RGS6 and RGS7 levels and thus regulates G-protein signaling in the retina.

## 2 Methods

### 2.1 Generation of Ate1 Conditional Knockout Mice

To obtain brain-specific Ate1 knockout mice, Ate1-floxed mice (Leu et al., 2009; Kurosaka et al., 2010) were crossed with the Jackson Laboratory strain B6.Cg-Tg(Nes-cre)1Kln/J, expressing Cre recombinase under Nesting promoter. Mice were maintained in C57BL6/129SVJ background in accordance with the University of Pennsylvania IACUC.

### 2.2 Immunohistochemistry and Data Processing

Retina collection and immunostaining was performed as previously described (Fina et al., 2021). Briefly, mice were dark adapted overnight and euthanized with a mixture containing ketamine/xylazine (300 *μ*g each per g body-weight). Immediately after the euthanasia, eyes were enucleated and the cornea removed under dim red-light. Eyeballs were fixed in the dark in 4% paraformaldehyde for 60 min, rinsed in phosphate buffer (PB) 3 times, cryoprotected overnight at 4*°C* in 0.1M PB containing 30% sucrose and then, with the lens removed, embedded in a mixture of two parts 20% sucrose in PB and one part optimal cutting temperature compound (Tissue Tek, Electron Microscopy Sciences, Hateld, PA, United States). Radial sections were stained with antibodies and imaged using a confocal laser scanning microscope (Olympus Fluoview 1,000, Center Valley, PA, United States) under an oil-immersion objective (Tummala et al., 2016). Littermate retinas from WT and KO mice were immunostained and imaged in parallel under the same conditions and settings. To compensate the exposure difference between imaging of retinal slides, each set of images from the same retinal slide was normalized by the non-specific signal in the outer nuclear layer (ONL).

For image quantification, a region of interest (ROI, of the same 6 × 6 *μ*m area size for WT and KO mice) was positioned on the lines drawn manually along the bands of stained starburst amacrine cell (SAC) processes in the inner plexiform layer (IPL), and the intensity measurement for each pixel was taken from the average z-stacks (of the same 0.3*μ*m/slice thickness for WT and KO mice) using MATLAB software (MathWorks, Natick, MA, United States). The pixel intensity was thresholded by the background—the non-specific signal in ONL. The average intensity of the pixels in the ROI was calculated for each section to represent the immunostaining level in IPL, averaged along the SAC bands for each section, and then averaged over 1 to 4 sections per retina.

### 2.3 mRNA Sequencing and Analysis

Tissue collection and processing was performed as previously described (Fina et al., 2021). Briefly, freshly excised mouse brains were flash-frozen in liquid nitrogen and ground in liquid nitrogen using mortar and pestle to obtain whole brain lysates. Total RNA was extracted using Trizol, and RNA sequencing (stranded TruSeq with Ribo-Zero, Illumina, San Diego, CA, United States) was used to quantify mRNA levels in the whole brain lysates of 3 littermate pairs of 3 month old mice and analyzed using STAR software package (Dobin et al., 2013).

### 2.4 Mass Spectrometry

Freshly excised mouse retinas, with the cornea and the lens removed, were flash-frozen in liquid nitrogen and ground up in liquid nitrogen to obtain retinal lysates. The retinal proteins were pulled down with phaloidin (manuscript in preparation).

For mass spectrometry, the resulting protein samples of 4 retinas of 6 month old mice were reduced with tris(2-carboxyethyl)phosphine (TCEP), alkylated with iodoacetamide, and digested with trypsin. Tryptic digests were analyzed using a 1.5 h LC gradient on the Thermo Q Exactive Plus mass spectrometer.

### 2.5 Mass Spectrometry Data Analysis

Data analysis was performed on the samples described above (processed as .raw files), as well as the original raw data files from public data sets (ProteomeXchange Consortium accession number PXD003441 (Zhao et al., 2016), PXD009909 (Harman et al., 2018), PXD014459 (Sze et al., 2021), and PXD023439 (Chen et al., 2021)), all of which are wild-type mice of C57BL6 backgrounds. The raw data files were processed using MaxQuant software (Version 1.6.7.0 for PXD014459 and 1.6.17.0 for the rest) from Max-Planck Institute for Biochemistry, Martinsried, Germany (Tyanova et al., 2016). To avoid mis-assignment of arginylation to a peptide, for each raw data file, we did two complete search runs, one without arginylation and one with arginylation modifications.

In the first run (without arginylation modifications), all searches were against the mouse protein reference UP000000589 and a locally compiled list of contaminants. In the second run, the “MS/MS first search” (used for mass recalibration in MaxQuant) was the same as the first run but the “MS/MS main search” and the “Second peptide search” were against the proteins of interest (Table 1). In addition, “Match between runs” and “Dependent peptides” were enabled in the second run. Consensus identification lists were generated with false discovery rates of 1% at protein, peptide and site levels. The two parameter .xml files and the two locally compiled. fasta files are included in the Supplementary Material. We excluded the “arginylated” peptides identified in the second run if they were from the scans which have at least one peptide identified in the first run. In addition, we also inspected MS/MS spectra to exclude “arginylated” peptides which could be mistaken for N-terminal or C-terminal arginine due to missed cleavage.

### 2.6 Antibodies and Constructs

Rabbit anti-G*β*5 (dilution 1:500) was a gift from Dr. C.K. Chen, Baylor College of Medicine, Houston, TX, United States; rabbit anti-RGS6/RGS7 (dilution 1:100) was a gift from Dr. T.G. Wensel, Baylor College of Medicine, Houston, TX, United States.

### 2.7 Experimental Design and Statistical Analysis

In the RNA sequencing and the immunostaining experiments, KO and WT littermate pairs were compared pairwise, and the p-values for these comparisons were calculated using paired Student’s t-test and paired Wilcoxon *t*-test (signed-rank test for median) on the 3 and 7 KO/WT pairs, respectively. For the immunostaining experiments, the average staining intensity of all samples on each slide was normalized to 1 and then the average staining intensity of all samples of each WT and KO pair was normalized to 1 in order to reduce the potential experimental variation.

## 3 Results and Discussions

### 3.1 Proteomic Screening for Arginylation of the Components of the G-protein Signaling Complex

We previously reported that the ON-bipolar responses and RGS7 protein levels are regulated by Ate1, however arginylation of RGS7 or in the components of the G-protein signaling complexes which give rise to ON-bipolar responses has never been directly demonstrated. To test whether any of these proteins are arginylated, we analyzed retina samples by mass spectrometry, and searched the results from LC-MS/MS runs for potential arginylation against a limited database including G-proteins, GPCRs, and RGS proteins (Table 1). These candidate proteins were chosen based on the existence of three well characterized G-protein signaling complexes in the retina. In rod and cone photoreceptors, rhodopsins and opsins are the GPCRs responding to photon stimulation in dim and bright light, respectively, and they activate G-proteins G*α*t1-G*β*1-G*γ*t1 and G*α*t2-G*β*3-G*γ*2 which are deactivated by RGS9 in the obligatory heterodimer with G*β*5L (Cowan et al., 1998; He et al., 1998). In ON-bipolar cells, glutamate released by photoreceptors activates the ON-bipolar cells’ GPCR, the metabotropic glutamate receptor 6 (mGluR6) receptor, which in turn activates the G*α*o-G*β*3-G*γ*13 proteins that can be deactivated by RGS7-G*β*5 and RGS11-G*β*5 (Mojumder et al., 2009; Chen et al., 2010; Zhang et al., 2010; Cao et al., 2012). We also included RGS6 in this screen, since it is the closest family member to RGS7 and is widely distributed in the retina, including prominent representation in the dendrites of cholinergic starburst amacrine cells (Voigt, 1986; Haverkamp and Wässle, 2000; Song et al., 2007).

To search for arginylation of these proteins, we used MaxQuant (Tyanova et al., 2016). To avoid mis-assignment, we first searched all MS/MS scans without arginylation modifications and then searched the unidentified scans with arginylation modifications, including potential addition of unmodified as well as mono- and di-methylated Arg. The search covered the G-proteins (Table 1, top) really well with the coverage ranging between 63 and 84 percent. However, the coverage for GPCRs was low, between 19 and 32 percent (Table 1, middle). This low coverage is likely related to the fact that GPCRs are tightly membrane-bound, and thus a majority of these proteins would be excluded from the soluble retina homogenate. The coverage of RGS and G*β*5, the GTPase accelerating proteins in the retina, varied a lot (Table 1, bottom). Most surprisingly, we found only one peptide for RGS11 in two MS/MS scans, despite the fact that RGS11 has similar molar amounts to RGS7 in the retina (Sarria et al., 2015).

This search revealed several putative arginylated sites, on G*α*t1, G*β*1, RGS6, and RGS7. These were the only G-protein and RGS family members identified. We found no instance of arginylation of the GPCRs. In the next sections, we detail the evidence for these arginylation sites.

### 3.2 Putative Arginylated Sites of G*α*t1

In outer segments of rod photoreceptors, the transducin G*α*t1 binds with G*β*1G*γ*t1 heterodimer in the dark. Light stimulation activates rhodopsin, which facilitates the formation of G*α*t1-GTP and hence promotes the dissociation of G*α*t1 from G*β*1*γ*t1 and rhodopsin. This activated G*α*t1 then interacts with the downstream phosphodiesterase (PDE6) and starts the phototransduction cascade.

**FIGURE 1:**
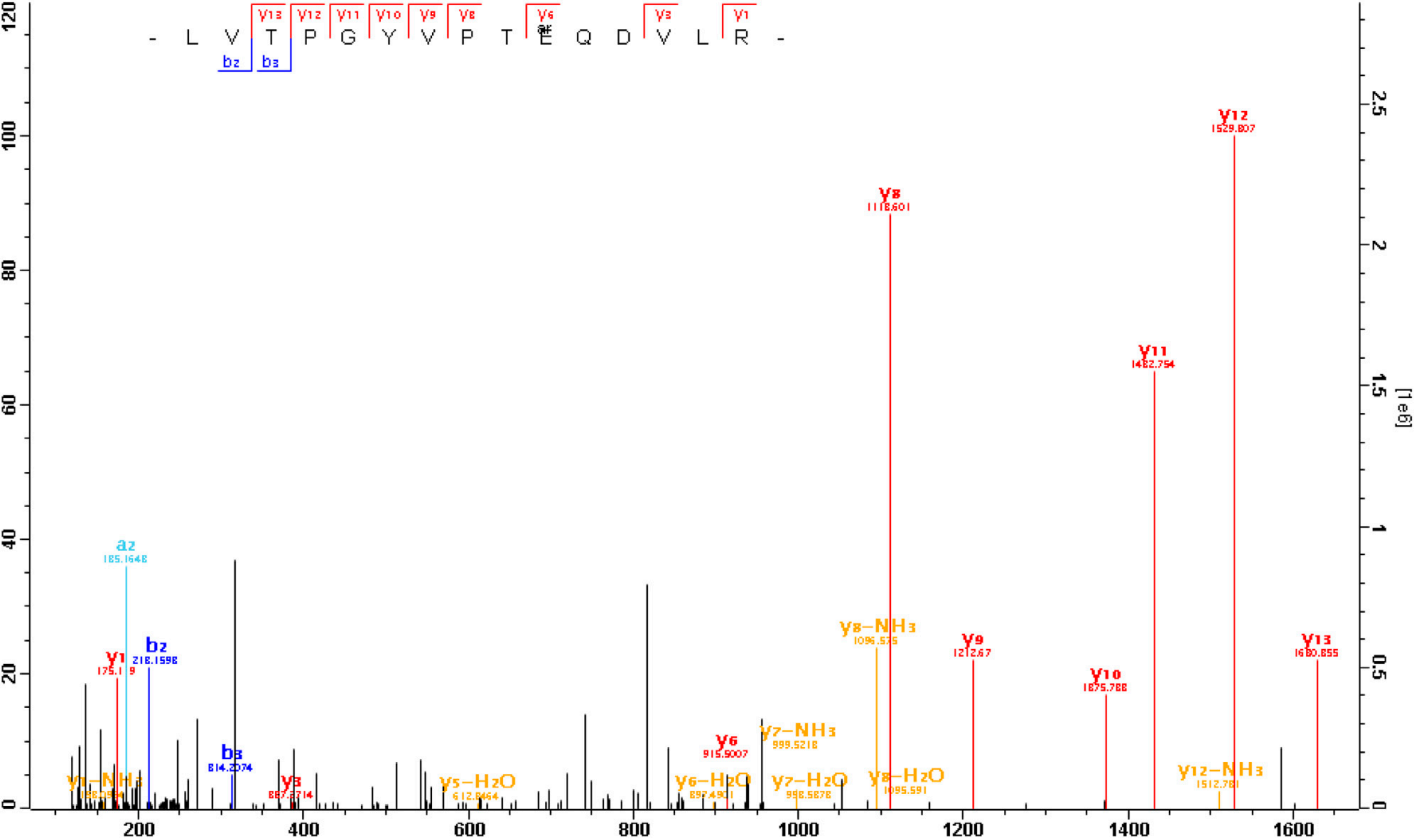
The MS/MS spectrum of an arginylated peptide of G*α*t1. The peptide LVTPGYVPT
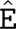
QDVLR or LVTPGYVPTEQD
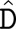
VLR (m/z = 615.007 3, mass error = − 0.3ppm, charge = 3) was arginylated at E167 or D169 of G*α*t1 (capped).

**FIGURE 2:**
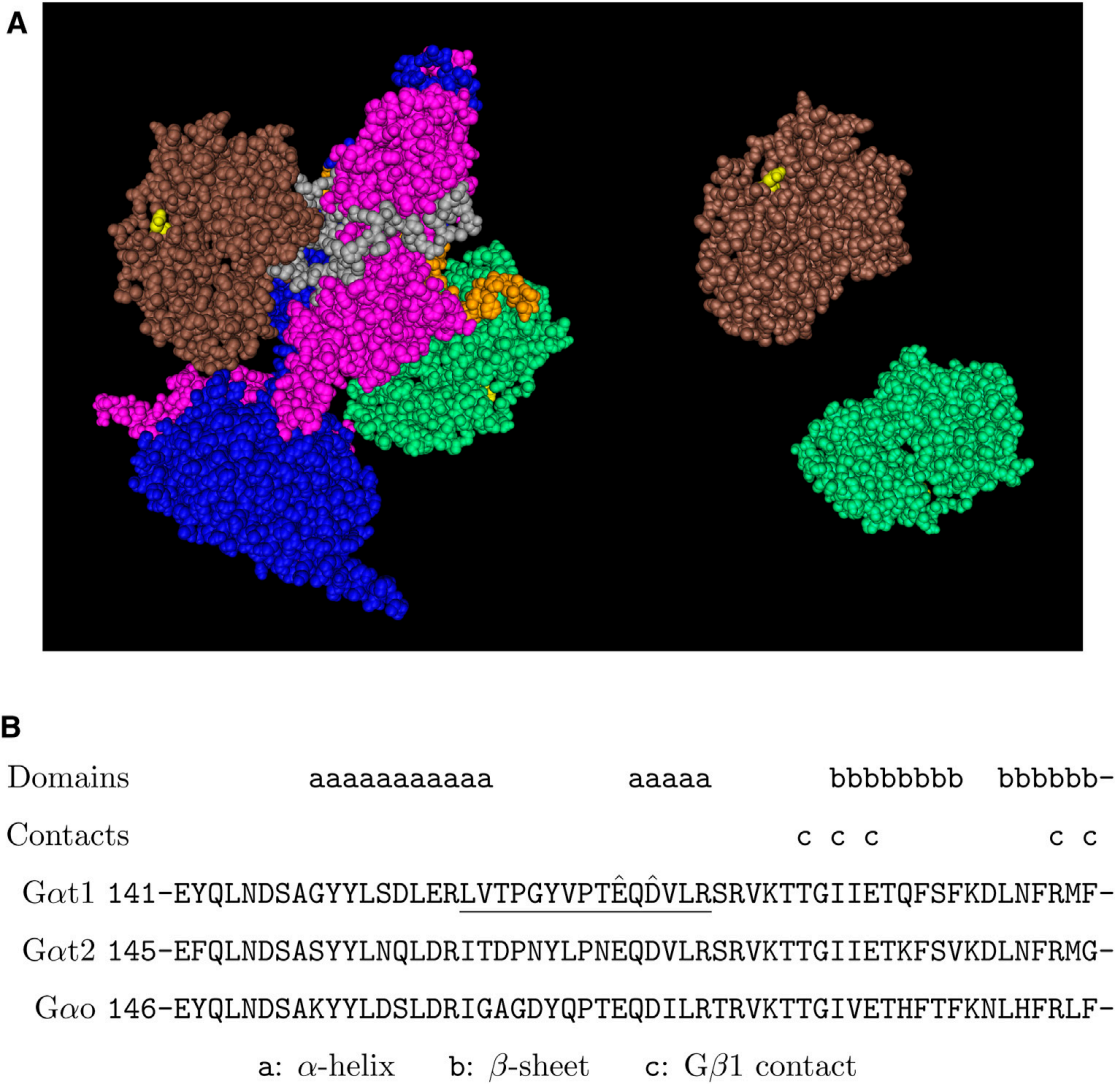
Putative arginylation sites of G*α*t1. **(A)** The 3D structure of 2 G*α*t1 in the complex with PDE6 based on the structure data 7JSN (Gao et al., 2020). Brown/green: G*α*t1, blue: PDE*α*, pink: PDE*β*, gray/orange: PDE*γ*, yellow: G*α*t1 E167. **(B)** The putative arginylation sites (capped), the peptide (underlined), and the surrounding regions are shown. The putative arginylation sites E167 and D169 are within the *α*-helical domain of six helices, near the beginning of the 6th *α*-helix which is connected by a linker region to the 2nd of the six-stranded *β*-sheets of the Ras-like GTPase domain (Lambright et al., 1996). These sites are shared between G*α*t1, G*α*t2, and G*α*o.

We found that G*α*t1 was arginylated at the site of E167 or D169 (Figure 1). This site is located in the middle of the protein and on the opposite side of the interface with PDE6 (Figure 2A). It is within the *α*-helical domain, near a linker with the *β*-sheets of the GTPase domain (Figure 2B). This site is also on the solvent-exposed surface of G*α*t1G*β*1G*γ*t1 heterotrimer in the GDP-bound state (Lambright et al., 1996), in a region of G*α*t1 *α*-helical domain, which interacts with G*β*1 when G*α*t1 is activated by rhodopsin, thus helping to provide an open route between the *α*-helical and GTPase domains and facilitating GDP and GTP exchange (Gao et al., 2019). This site is shared between G*α*t1, G*α*t2, and G*α*o (Figure 2B). Arginylation of this site can affect the rate of GDP-GTP exchange of these G*α* proteins and the G-protein signaling in rods, cones, and ON-bipolar cells.

### 3.3 Putative Arginylated Sites of G*β*1

In the outer segments of the rods, G*β*1 is the important link between G*α*t1 and G*γ*t1. Each of G*α*t1 and G*γ*t1 has a membrane domain, which is not strong enough to anchor G*α*t1 or G*β*1G*γ*t1 separately to the disk membrane, but both of them together anchor the heterotrimer in the GDP-bound inactive state. Recently, it has been proposed to play an important role in facilitating GDP-GTP exchange during G*α*t1 activation (Gao et al., 2019). G*β*1 is also found in the synaptic terminals of the rods (Peng et al., 1992). Interestingly, G*β*1, when translocated to the synaptic terminals of the rods during light adaptation (Whelan and McGinnis, 1988; Sokolov et al., 2002) facilitates the synaptic transmission to ON-bipolar cells (Majumder et al., 2013).

**FIGURE 3:**
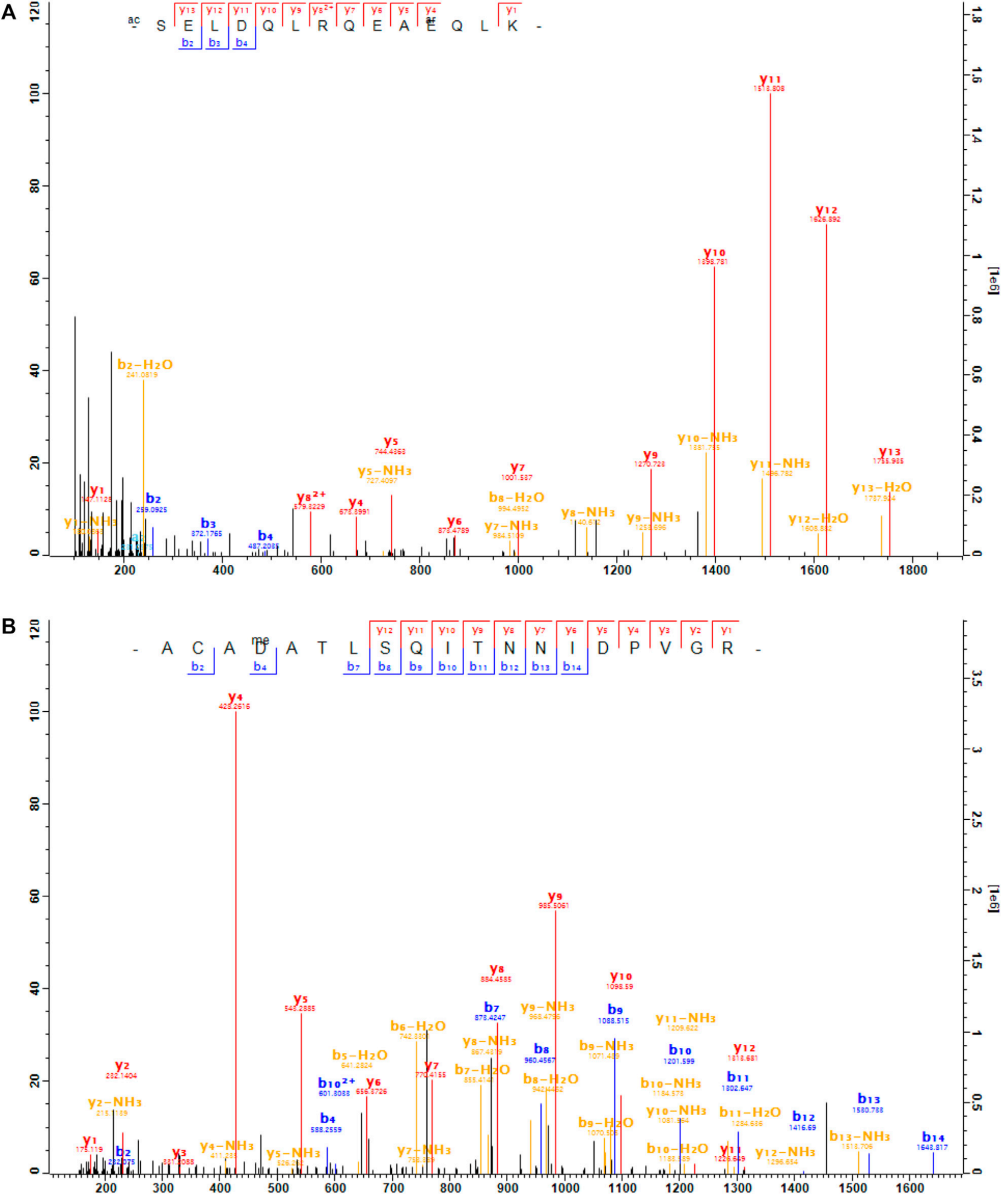
The MS/MS spectra of the arginylated peptides of G*β*1. **(A)**, the peptide SELDQLRQEA
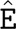
QLK (m/z = 628.997 3, mass error = 0.4ppm, charge = 3) was arginylated at E12 of G*β*1 (capped). **(B)**, the peptide ACA
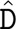
ATLSQITNNIDPVGR (m/z = 729.370 9, mass error = − 4ppm, charge = 3) was methyl-arginylated at D27 of G*β*1 (capped).

**FIGURE 4:**
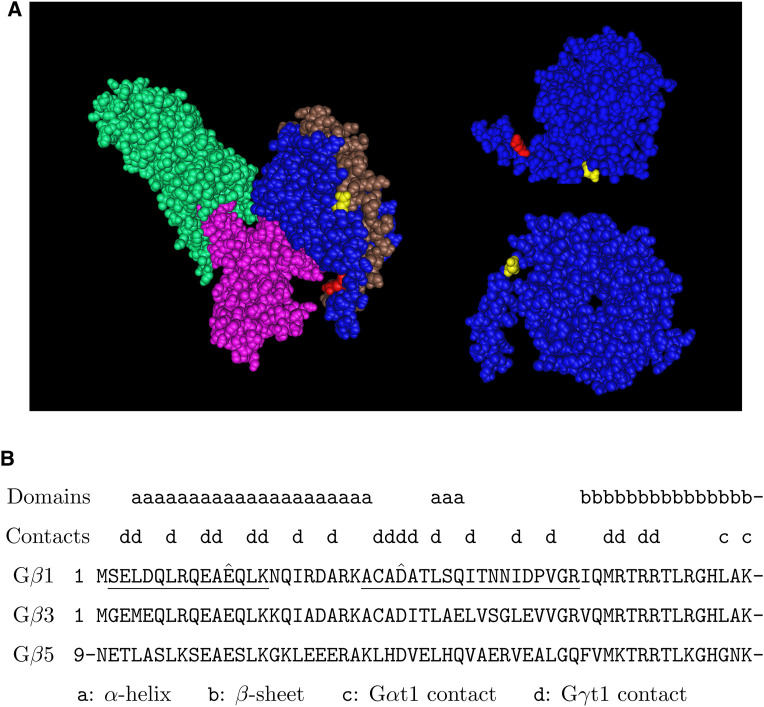
Putative arginylation sites of G*β*1. **(A)** The 3D structure of G*β*1 in the G*α*t1-G*β*1-G*γ*t1 heterotrimer in complex with rhodopsin based on the structure data 6OYE (Gao et al., 2019). Blue: G*β*1, brown: G*γ*t1, pink: G*α*t1, green: rhodopsin, red: G*β*1 E12, yellow: G*β*1 D27. **(B)** The putative arginylation sites (capped), the peptides (underlined), and the surrounding regions are shown. The putative arginylation sites are on the N-terminal directly proceeding the first *β*-sheet of the seven WD40 domains (Sondek et al., 1996). These sites are shared between G*β*1, G*β*3, and G*β*5.

We found that G*β*1 was arginylated at two sites: E12 and D27 (Figure 3). These sites are located near the N-terminus of the protein before the seven-bladed *β*-propellers (WD40 domains) and in particular D27 is at the junction of the G*β*1 N-terminus, seven-blades, and G*γ* (Figure 4A). These sites are located within a region of high homology and is conserved between G*β*1, G*β*3, and G*β*5 (Figure 4B). Based on this homology, we propose that these sites can also be arginylated in G*β*3 and possibly in G*β*5. G*β*3 bound with G*γ*2 plays a similar role in the cones as G*β*1 in the rods. G*β*3 is also found in the synaptic terminals of the cones (Peng et al., 1992). Arginylation of G*β*1 and G*β*3 can potentially affect the phototransduction in the outer segments and the signal transmission to ON-bipolar cells (OBCs) at the synaptic terminals of rods and cones, respectively. In addition, G*β*3 in complex with G*γ*13 in OBCs facilitates the post-synaptic light-on responses of both rod-OBCs and cone-OBCs (Dhingra et al., 2012; Ramakrishnan et al., 2015) and thus arginylation of G*β*3, if it happens, can potentially affect post-synaptic responses to both rod and cone signals.

G*β*5 is a special G*β* existing as an obligatory heterodimer with all R7 family (RGS6, RGS7, RGS9, RGS11) proteins (Cabrera et al., 1998; Liang et al., 2000; Witherow et al., 2000; Zhang and Simonds, 2000). It has 2 splice variants, G*β*5 (short) and G*β*5L, which includes additional 42 N-terminal amino acid residues added to the G*β*5 (short) sequence. In the retina, the G*β*5L isoform forms an obligatory heterodimer with RGS9 (short), and the G*β*5 isoform forms obligatory heterodimers with RGS6, RGS7, or RGS11. Arginylation of G*β*5 can affect all the G-protein signaling in the retina, predominately regulated by R7 family proteins.

### 3.4 Putative Arginylation Sites of RGS6 and RGS7

RGS6 is the closest family member to RGS7 and presumably shares many structural and functional properties with RGS7. We have previously found that the deletion of Arginyltransferase 1 (Ate1) in the retina leads to a prominent increase in the levels of RGS7 and its obligatory binding partner G*β*5 in the dendritic processes of ON-bipolar cells (Fina et al., 2021), but in that work we found no direct evidence of arginylation.

**FIGURE 5:**
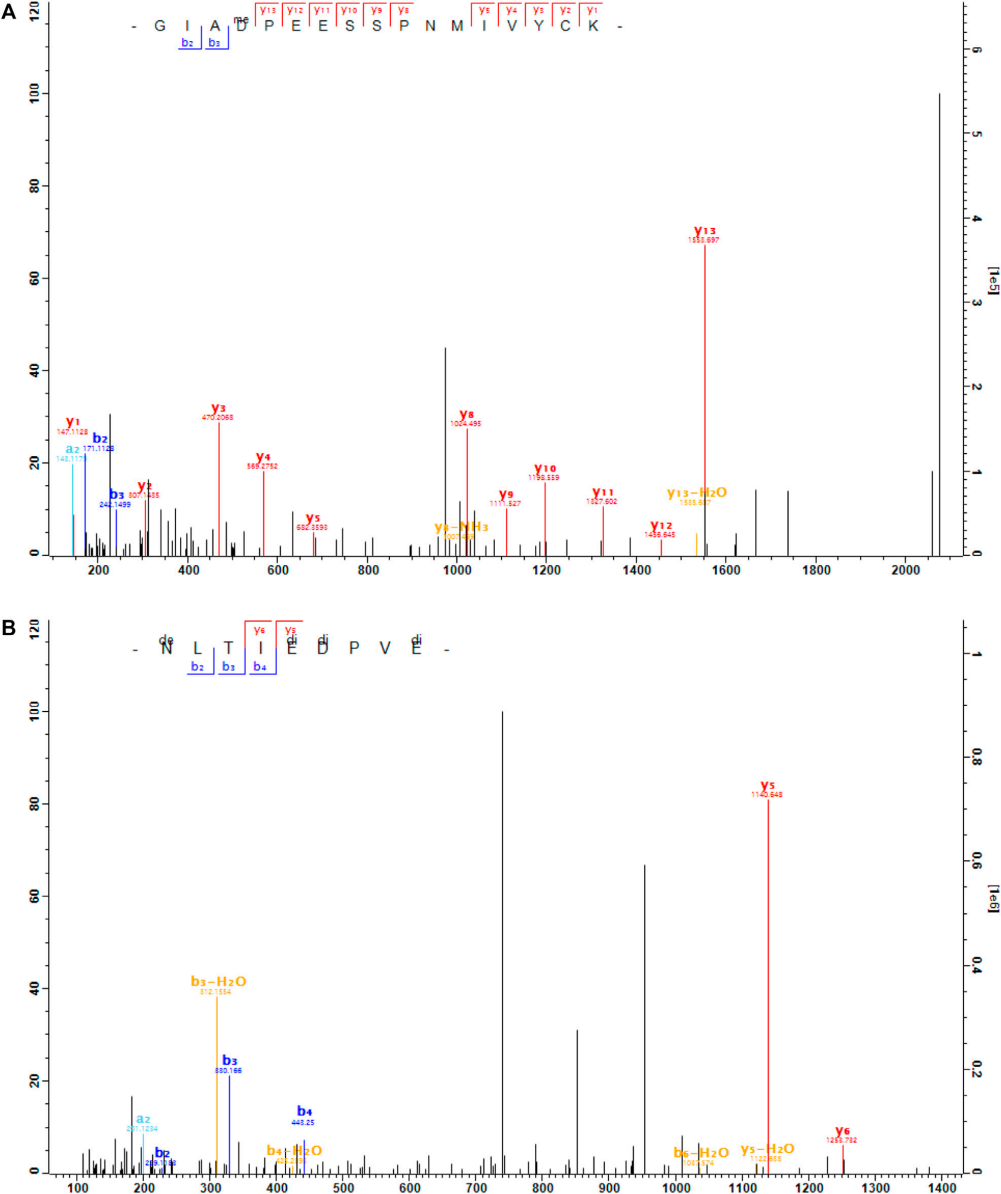
The MS/MS spectra of the arginylated peptides of RGS6 and RGS7. **(A)**, the peptide GIAD
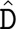
PEESSPNMIVYCK (m/z = 1,040.495 5, mass error = − 5ppm, charge = 2) was methyl-arginylated at D15 of RGS6 (capped). **(B)**, the peptide NLTI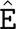 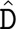
PV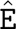 (m/z = 791.949 2, mass error = 2ppm, charge = 2) was dimethyl-arginylated at E73, D74, and E77 of RGS7 (capped).

**FIGURE 6:**
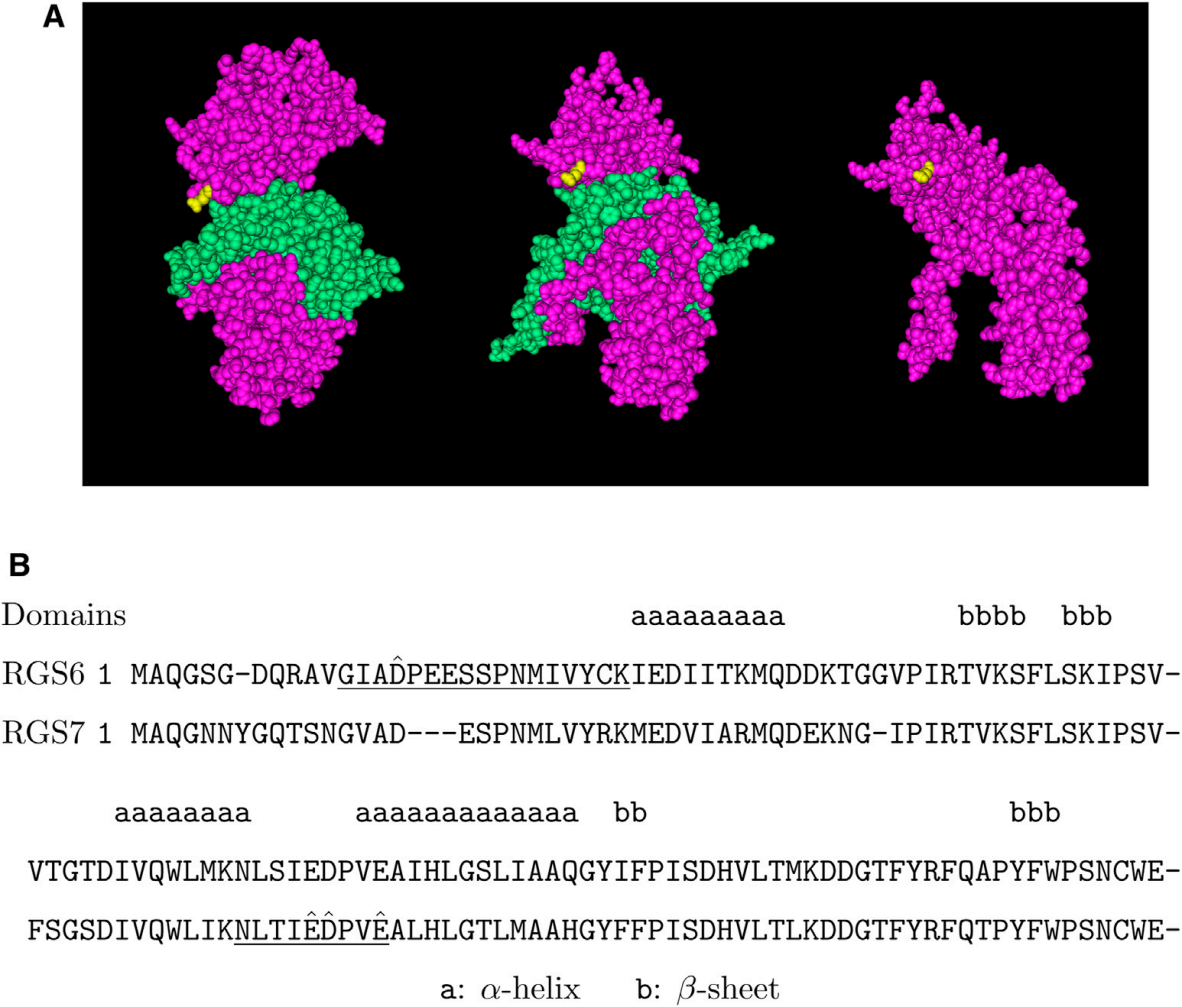
Putative arginylation sites of RGS6 and RGS7. **(A)** The 3D structure of RGS7 in the RGS7-G*β*5 heterodimer based on the structure data 6N9G (Patil et al., 2018). Pink: RGS7, green: G*β*5, yellow: RGS7 E73. **(B)** The putative arginylation sites (capped), the peptides (underlined), and the surrounding regions are shown with RGS6 and RGS7 alignment (Altschul et al., 1997). The putative arginylation sites are near/on the DEP domain, one in front of the 1st *α*-helix and three between the 2nd and 3rd *α*-helices in the secondary structures (Wong et al., 2000). These sites are shared between RGS6 and RGS7.

Here, we found that RGS6 was arginylated at the site of D15 and RGS7 was arginylated at three sites: E73, D74 and E77 (Figure 5). These sites are located in the N-terminal region which has the membrane targeting DEP domain. These sites are conserved between RGS6 and RGS7, and the surrounding regions are highly homologous between RGS6 and RGS7 (Figure 6). Thus, it is likely that these sites are arginylated in both RGS6 and RGS7, and that this arginylation can directly contribute to an increase of their levels in synaptic processes of starburst amacrine cells and ON-bipolar cells, respectively.

This is the first evidence that R7 family RGS proteins are arginylated on side chains, in contrast to the previous finding that R4 family RGS proteins are arginylated on N-termini (Lee et al., 2005).

### 3.5 RGS6 and RGS7 Proteins Are Enriched in the G-protein Signaling Complexes in the Absence of Arginylation

To test whether abolishment of arginylation affects the level of RGS6 and RGS7 proteins in the G-protein signaling complexes, we compared the RGS6 and RGS7 protein levels in retinal synaptic processes of the wild-type (WT) and the conditional Ate1 knockout (KO) mice. In the KO mice, Ate1 deletion is driven by neuron-specific Nestin promoter, occurring in the entire nervous system during embryogenesis (Wang et al., 2017a; Wang et al., 2017b). ATE1 protein level in these mice is greatly reduced in most retinal neurons (Fina et al., 2021).

**FIGURE 7:**
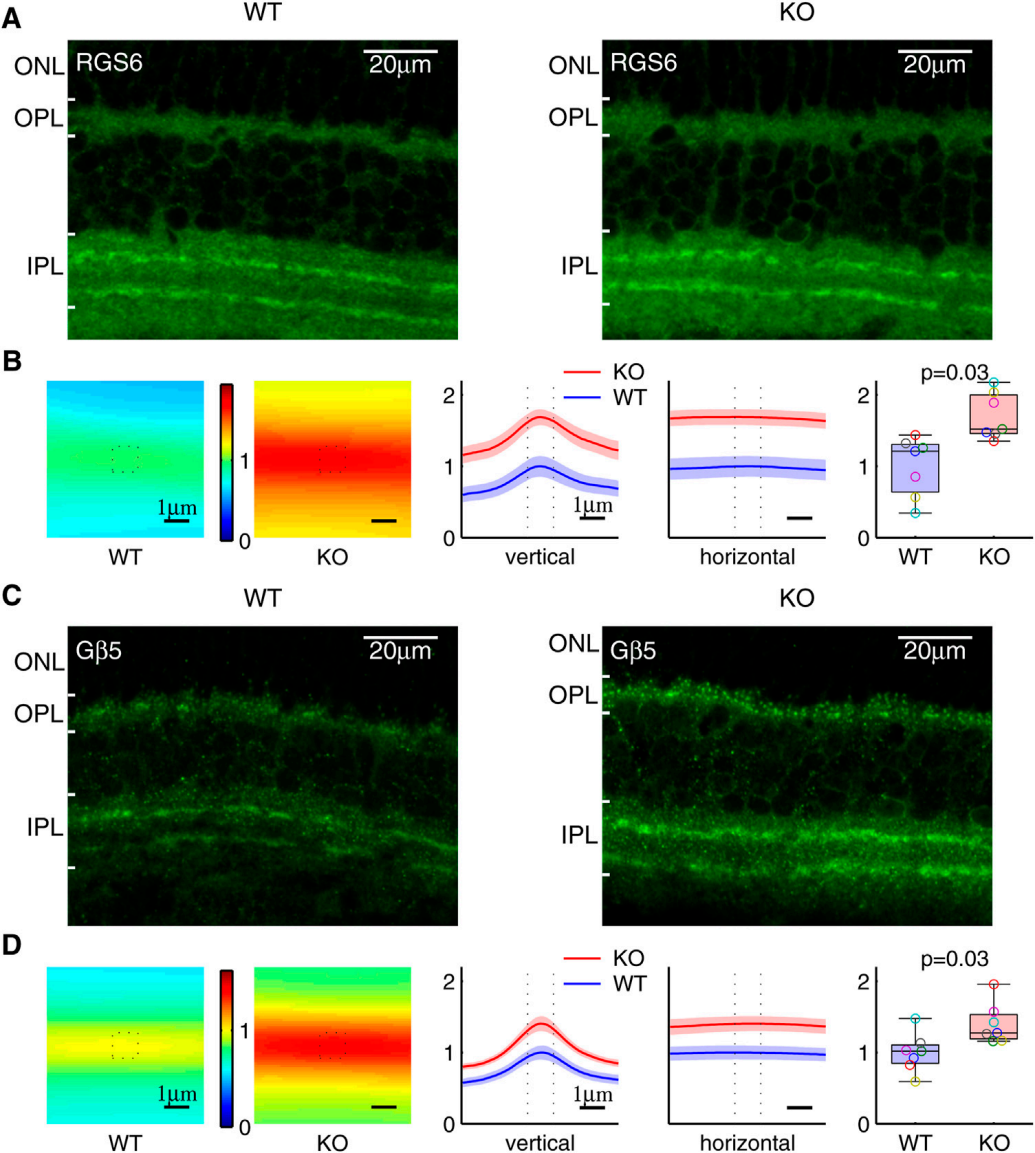
Increased RGS6 and G*β*5 protein level in the synaptic processes of Ate1 knockout (KO) mouse. **(A)** Immunohistochemistry of retinas stained with anti-RGS6. Both the wild-type (WT) and KO retinas have clear staining of two distinct bands of synaptic processes in the inner plexiform layer (IPL). **(B)** The RGS6 staining intensities averaged along the bands are shown on the left. The values along the vertical and horizontal middle lines are plotted in the middle with the SEM in shaded colors. The dotted lines mark the central 1.1 *μm* region surrounding the maxima. In the box plot on the right, the total sum at the central region shows a significant increase of RGS6 proteins in the synaptic processes of KO mice. **(C)** and **(D)** Immunohistochemistry of retina stained with anti-G*β*5 show the same pattern and the results as **(A)** and **(B)** for RGS6. Each pair of data points of the same color represents a WT/KO littermate pair (n = 7). The p-values are from paired Wilcoxon *t*-test (signed-rank test for median). The maximum length of the whiskers are 1.5 times of the inter quartile range. Note: the anti-G*β*5 and anti-RGS6 also stained puncta in ON-bipolar dendrites in the outer plexiform layer (OPL)—the latter due to a cross interaction with RGS7; however, its staining in IPL represents RGS6 only since RGS7 is not present in IPL (Song et al., 2007). ONL: the outer nuclear layer.

**FIGURE 8:**
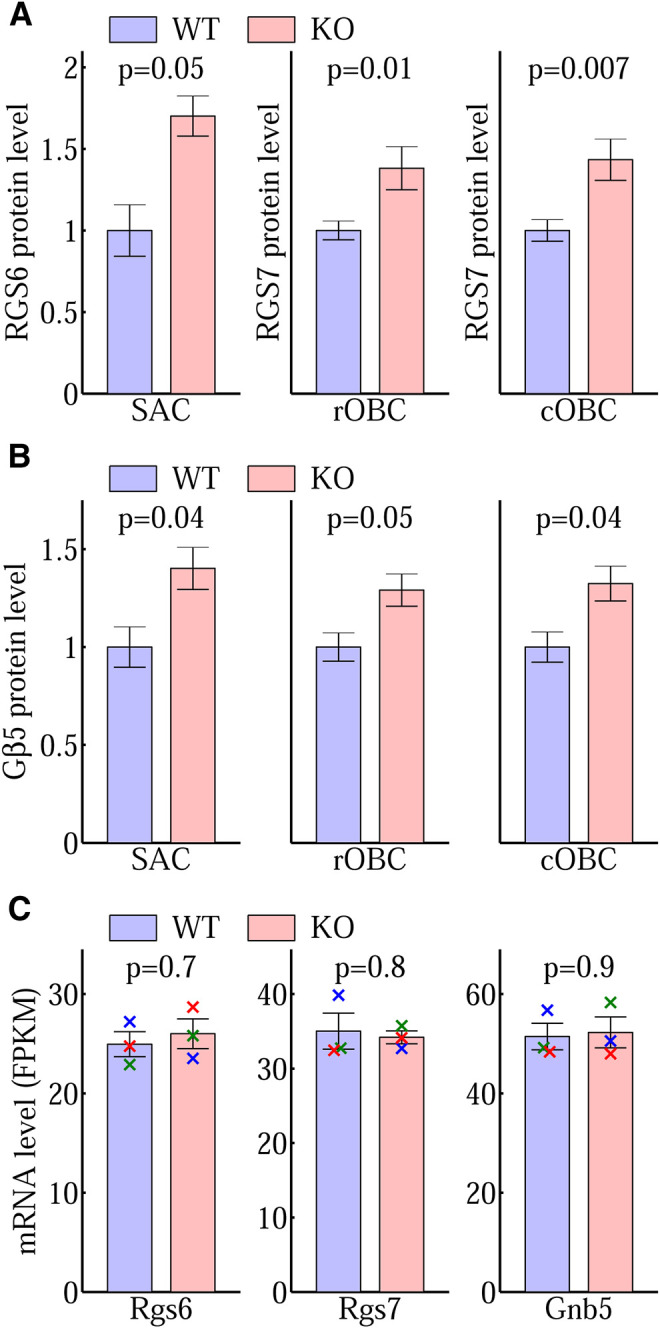
Post-translational changes of RGS6, RGS7, and G*β*5. Immunostaining quantification of **(A)** the protein levels of RGS6 in SAC and RGS7 in rOBC and cOBC synaptic processes and **(B)** the corresponding G*β*5 levels demonstrate a significantly higher level of these proteins in the Ate1 knockout (KO) than the wild-type (WT) mice. SAC: starburst amacrine cell. rOBC: rod ON-bipolar cell. cOBC: cone ON-bipolar cell. In **(A)** and **(B)**, the bar plots for SAC are based on the same data of the box plots of Figure 7 and the bar plots for rOBC and cOBC are based on the data of Fina et al. (2021). The p-values are from paired Student’s t-test and the error bars represent SEM. The differences between KO and WT were also evaluated with a non-parametric statistical hypothesis test (Figure 7 for RGS6 and G*β*5 in SAC; Fina et al. (2021) for RGS7 and G*β*5 in rOBC and cOBC), which confirmed the conclusions in **(A)** and **(B)**. **(C)** The Rgs6, Rgs7, and Gnb5 (short) mRNA encoding RGS6, RGS7, and G*β*5 proteins in the mouse brains from WT and KO littermates, expressed in Fragments Per Kilobase of transcript per Million mapped reads (FPKM) from RNA sequencing. There is no significant difference between WT and KO. Each pair of data points of the same color represents a WT/KO littermate pair (*n* = 3). The p-values are from paired Student’s t-test and the error bars represent SEM. The p-values from non-parametric statistical hypothesis test (paired Wilcoxon *t*-test) (0.8, 1, and 1, respectively) also shows no significant difference in **(C)**.

RGS6 and RGS7 in the retina are prominently expressed in the synaptic processes of starburst amacrine cells in the inner plexiform layer (IPL) and ON-bipolar cells in the outer plexiform layer (OPL), separately (Song et al., 2007). In agreement with previous studies, our immunohistochemistry staining showed that RGS6 was prominently enriched in the starburst amacrine cell (SAC) processes, which are localized within two distinct bands in the IPL (Figure 7A). We used the total sum within the 1.1*μm* × 1.1 *μm* window, centered and averaged along the SAC bands, to quantify the RGS6 protein level in the synaptic processes. The RGS6 protein level was significantly higher in KO than in WT (Figure 7B). Consistent with that, RGS6’s obligatory binding partner G*β*5 was also enriched in the two distinct bands in the IPL (Figure 7C) and its level was significantly higher in KO than in WT (Figure 7D). In addition, in a separate study, we showed that RGS7 and G*β*5 proteins levels were significantly higher in KO than in WT dendritic processes of both rod- and cone-OBCs in the OPL (Fina et al., 2021). These results are summarized in Figures 8A,B.

### 3.6 Post-Translational Changes of RGS6 and RGS7 Proteins

It is interesting to know if the observed increase of RGS6 and RGS7 proteins at the synaptic processes are associated with an overall increase of their levels in the retina. In our previous study, we used Western blots to show that in total retina extracts RGS7 levels increased in Ate1 knockout, but G*β*5 did not (Fina et al., 2021). Thus, since RGS6 is the dominant binding partner with G*β*5 in the retina (Shim et al., 2012) and they form an obligatory complex *in vivo*, it would be highly unlikely for the total RGS6 in the retina to increase significantly in the absence of arginylation without simultaneously causing G*β*5 to do the same.

We compared the mRNA levels of Rgs6, Rgs7, and Gnb5 of Ate1 KO and WT mice. There was no significant difference for any of them (Figure 8C). This indicates that the changes of the corresponding protein levels and/or distributions are most likely post-translational.

It is conceivable that the overall increase of RGS7 protein level causes its increase at ON-bipolar dendritic processes. However, it cannot be the case for RGS6. Its redistribution to SAC synaptic processes upon arginylation could be due, e.g., to the fact that arginylation of RGS6 and/or other proteins in SAC could contribute to its targeting to the membrane and the synapses. It is known that light exposure leads to redistribution of G-proteins in the rods from the outer segments to the inner segments and the rod terminals (Whelan and McGinnis, 1988; Sokolov et al., 2002). Our ongoing study also shows that RGS7 levels at the synapses change significantly depending on the synaptic activity. Since Ate1 knockout increases the activities of ON-bipolar cells and down-stream neurons (Fina et al., 2021), RGS6 can undergo activity-dependent redistribution. Similar mechanisms could also be at work with RGS7. One possibility is that Ate1 knockout changes ON-bipolar activities pre-synaptically through G*β*1.

G*β*1 plays an important role in the rods. In addition to the rising phase of light responses in the outer segments, it also facilitates the synaptic transmission to OBCs (Majumder et al., 2013). Here, we presented evidence that G*β*1 is arginylated. However, we did not observe any significant change of the rising phase of the light responses of the rods after deletion of Ate1 (Fina et al., 2021). In our current ongoing project, we also did not see any obvious change in G*β*1 in the outer segments after Ate1 deletion (manuscript in preparation). It should be noted that lack of changes we have seen so far in G*β*1 does not exclude a possible effect of G*β*1 at the pre-synaptic terminals of photoreceptors, in contact with On-bipolar cell dendritic processes. It would be very interesting to address this possibility in a follow up study.

According to prior studies, RGS6 is not implicated in G-protein signaling in photoreceptors or ON-bipolar cells, where the major players are RGS9 (in the photoreceptors) and RGS7/RGS11 (in the ON-bipolar cells). In our experiments, the strongest RGS6 staining and the significant change due to Ate1 deletion, by far, was observed in the synaptic processes of starburst amacrine cells. This suggests that RGS6 likely plays an arginylation-dependent role in G-protein signaling in these cells. Elucidating this role is a very interesting future direction, and we hope our present findings will help future research to reveal the functional role of RGS6 in the retina.

## 4 Summary

This work represents the first comprehensive analysis of arginylation of the components of G-protein signaling in the retina, including GPCR, G-proteins, and RGS proteins. We find putative arginylation sites on the aspartate and glutamate side chains of G*α*t1, G*β*1, RGS6, and RGS7, and no N-terminal arginylation among the proteins involved in the G-protein signaling in the retina. While, due to probabilistic nature of mass spectrometry analysis of post-translational modifications, as well as potential alteration of protein stability or intracellular localization of proteins after arginylation, it is still possible that other proteins in the retina are also arginylated, the targets identified here are likely more prominent and abundant.

Our results provide concrete evidence that arginylation targets proteins involved in G-protein signaling in the retina, and extend a mechanistic foundation to our previous findings that arginylation regulates retina responses in vision. We show here that abolishment of arginylation leads to an increase in RGS6 and RGS7 protein levels, suggesting that this mechanism can directly regulate the events downstream of RGS. Taken together, our findings demonstrate that arginylation regulates G-protein responses in the retina by directly targeting several key players in this signaling cascade.

## Data Availability

The datasets presented in this study can be found in online repositories. The names of the repository/repositories and accession number(s) can be found in the article/Supplementary Material.
